# Supplementary material for: Thermal spectrometer for superconducting circuits
Source: Nat Commun. 2025 May 13;16:4435. doi: 10.1038/s41467-025-58919-8 (PMC12075477; doi:10.1038/s41467-025-58919-8)
Supplement: Supplementary file 1 — Supplementary Information [file 41467_2025_58919_MOESM1_ESM.pdf]

Supplementary Information
Thermal spectrometer for superconducting circuits

Christoforus Dimas Satrya[1],* Yu-Cheng Chang[1], Aleksandr S. Strelnikov[1], Rishabh Upadhyay[1,2], Ilari K. Mäkinen[1], Joonas T. Peltonen[1], Bayan Karimi[1,3], and Jukka P. Pekola[1]
[1] *Pico group, QTF Centre of Excellence, Department of Applied Physics, Aalto University School of Science, P.O. Box 13500, 00076 Aalto, Finland*
[2] *VTT Technical Research Centre of Finland Ltd, Tietotie 3, 02150 Espoo, Finland and*
[3] *Pritzker School of Molecular Engineering, University of Chicago, Chicago IL 60637, USA*
(Dated: April 2, 2025)

I. POWER TRANSMISSION FROM CIRCUIT THEORY

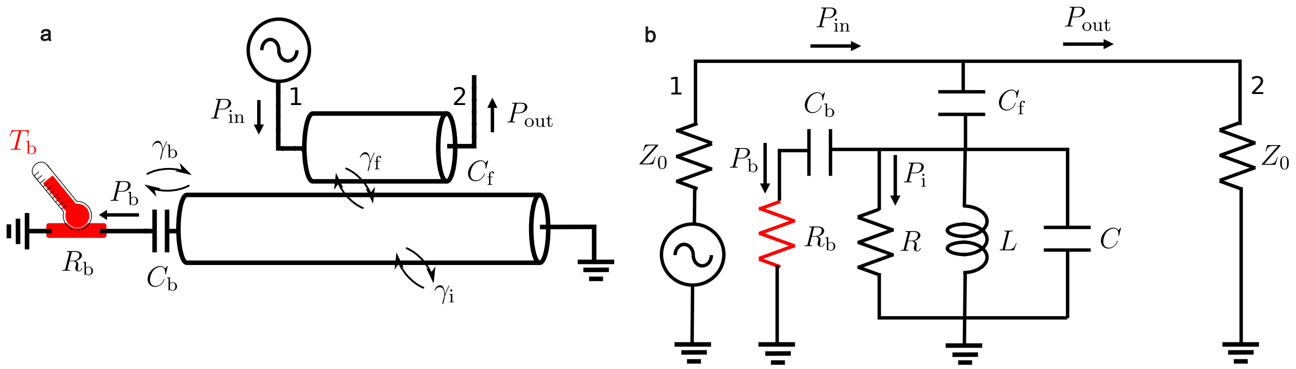

Figure S 1. **Principle of characterizing resonator by using a bolometer**. **a**, The photons in the resonator are excited due to the input microwave tone $P_{\rm in}$ that is injected to the nearby feedline. Temperature rise of the bolometer, due to photon leakage from the resonator, contains information of the total loss rate of the photons $2\pi\gamma_{\rm t}$. **b**, Equivalent lumped circuit. The bolometer is heated up due to the dissipation power of $P_{\rm b}$ transmitted to the absorber $R_{\rm b}$. The magnitude of $P_{\rm b}$ depends on the impedance of the other circuit elements of $R, L,$ and C. $P_{\rm i}$ is power dissipation at internal bath.

The circuit of the experimental setup is shown in Fig. 1a. Steady power $P_{\rm in}$ at a single frequency f is generated at the input port 1 and the dissipated power $P_{\rm b}$ on resistor $R_{\rm b}$ is simultaneously measured. Here, with circuit model, we will derive the power transmission coefficient $|S(f)|^2$ defined by the equation:

$$P_{\rm b}(f) = |S(f)|^2 P_{\rm in}. \tag{1}$$

Since the magnitude of $P_{\rm in}$ is frequency independent, the coefficient $|S(f)|^2$ determines the frequency dependency in $P_{\rm b}(f)$.

Close to its resonance frequency (f_0), the $\lambda/4$-resonator can be approximated by a lumped parallel LRC-circuit (shown in Fig. 1b) with the parameters [1]:

$$C = \frac{\pi}{4\omega_0 Z_0} = 1/8Z_0 f_0 \tag{2}$$

$$L = \frac{1}{\omega_0^2 C} = 2Z_0/\pi^2 f_0 \tag{3}$$

$$R = Z_0/\alpha l, \tag{4}$$

* christoforus.satrya@aalto.fi

where l is the length of the resonator, $\omega_0 = 2\pi f_0$ is the resonant frequency of the approximated mode, Z_0 is the characteristic impedance of the transmission line and α is the attenuation constant.

By applying the results from the Appendix A of Ref. [2], the power transmission coefficient can then be written as

$$|S|^2 = \frac{2\mathrm{Re}(1/Z_\mathrm{f})\mathrm{Re}(1/Z_\mathrm{b})}{|1/Z_\mathrm{f} + 1/Z_\mathrm{b} + j\omega C + 1/j\omega L + 1/R|^2}, \tag{5}$$

where we have defined the terminating impedances

$$Z_\mathrm{f} = Z_0/2 + \frac{1}{j\omega C_\mathrm{f}} \tag{6}$$

and

$$Z_\mathrm{b} = R_\mathrm{b} + \frac{1}{j\omega C_\mathrm{b}}. \tag{7}$$

Furthermore, to simplify the expression, we define parallel effective resistance and capacitance

$$1/Z_k = 1/R_k^\mathrm{eff} + j\omega C_k^\mathrm{eff}, \tag{8}$$

for $k = \mathrm{f}, \mathrm{b}$, which transforms the transmission formula into

$$|S|^2 = \frac{2(1/R_\mathrm{f}^\mathrm{eff})(1/R_\mathrm{b}^\mathrm{eff})}{|j\omega C_\mathrm{t} + 1/j\omega L + 1/R_\mathrm{t}|^2}, \tag{9}$$

where $C_\mathrm{t} = C + C_\mathrm{f}^\mathrm{eff} + C_\mathrm{b}^\mathrm{eff}$, and $1/R_\mathrm{t} = 1/R + 1/R_\mathrm{f}^\mathrm{eff} + 1/R_\mathrm{b}^\mathrm{eff}$ which makes the problem equivalent to calculating the transfer coefficient between two resistors $R_\mathrm{f}^\mathrm{eff}$ and $R_\mathrm{b}^\mathrm{eff}$ connected with a LCR circuit with its capacitance transformed into C_t and resistance R_t. The inverse total quality factor ($1/Q_\mathrm{t}$) of this combined circuit reads

$$\begin{aligned} 1/Q_\mathrm{t} = \frac{1}{\omega_0' C_\mathrm{t} R_\mathrm{t}} &= \frac{1}{\omega_0' C_\mathrm{T} R} + \frac{1}{\omega_0' C_\mathrm{T} R_\mathrm{f}^\mathrm{eff}} + \frac{1}{\omega_0' C_\mathrm{T} R_\mathrm{b}^\mathrm{eff}} \\ &= 1/Q_\mathrm{i} + 1/Q_\mathrm{f} + 1/Q_\mathrm{b}, \end{aligned} \tag{10}$$

with shifted resonance frequency $\omega_0' = 1/\sqrt{LC_\mathrm{t}}$. Index i,f and b here is quality factor for internal, feedline, and bolometer respectively. Moreover, to make further simplifications, in the case where

$$C_\mathrm{f}^\mathrm{eff}, C_\mathrm{b}^\mathrm{eff} \ll C, \tag{11}$$

we can write the quality factor as

$$1/Q_k = \frac{1}{\omega_0 C R_k^\mathrm{eff}}. \tag{12}$$

In particular, in our specific circuit case we have

$$Z_k = 1/(j\omega C_k) + R_k, \tag{13}$$

which yields

$$1/Z_k = \frac{R_k}{R_k^2 + \frac{1}{(\omega C_k)^2}} + \frac{j/(\omega C_k)}{R_k^2 + \frac{1}{(\omega C_k)^2}} = 1/R_k^\mathrm{eff} + j\omega C_k^\mathrm{eff}, \tag{14}$$

resulting in the Norton equivalent relations:

$$R_k^\mathrm{eff} = R_k + \frac{1}{R_k(\omega C_k)^2} \tag{15}$$

$$C_k^\mathrm{eff} = \frac{1/(\omega^2 C_k)}{R_k^2 + \frac{1}{(\omega C_k)^2}}. \tag{16}$$

Therefore, we can calculate the quality factor as

$$\begin{aligned} Q_k = \omega_0 C R_k^{\text{eff}} &= \omega_0 C R_k + \omega_0 C \frac{1}{R_k(\omega_0 C_k)^2} \\ &= \omega_0 C R_k + \frac{\sqrt{L/C}}{R_k}(C/C_k)^2 \\ &= \omega_0 C R_k + \frac{Z_{\text{LC}}}{R_k}(C/C_k)^2 \approx \frac{Z_{\text{LC}}}{R_k}(C/C_k)^2, \end{aligned} \tag{17}$$

where $Z_{\text{LC}} = \sqrt{L/C}$, and $k = \text{f}, \text{b}$ for feedline and bolometer respectively.

Finally, to connect the transmission coefficient to the quality factors, with Eq. 12 let's rewrite Eq. 9 in the form

$$\begin{aligned} |S|^2 &= \frac{2(\omega_0 C)^2 (1/Q_{\text{f}})(1/Q_{\text{b}})}{|\omega_0 C(1/Q_{\text{t}}) + j\omega_0 C[\omega/\omega_0 - 1/(\omega\omega_0 LC)]|^2} \\ &= \frac{2(1/Q_{\text{f}})(1/Q_{\text{b}})}{|(1/Q_{\text{t}}) + j(\omega/\omega_0 - \omega_0/\omega)|^2} \\ &= \frac{2(1/Q_{\text{f}})(1/Q_{\text{b}})}{(\omega/\omega_0 - \omega_0/\omega)^2 + (1/Q_{\text{t}})^2} \\ &\approx \frac{2(1/Q_{\text{f}})(1/Q_{\text{b}})}{4(\omega/\omega_0 - 1)^2 + (1/Q_{\text{t}})^2} \\ &= \frac{(1/2Q_{\text{f}})(1/Q_{\text{b}})}{(\omega/\omega_0 - 1)^2 + (1/2Q_{\text{t}})^2}, \end{aligned} \tag{18}$$

where $1/Q_{\text{t}} = 1/Q_{\text{i}} + 1/Q_{\text{f}} + 1/Q_{\text{b}}$ is total inverse quality factor, $Q_{\text{i}} = \omega_0 C R$ is internal quality factor, $Q_{\text{f}} = \frac{2Z_{\text{LC}}}{Z_0}(C/C_f)^2$ is feedline quality factor, and $Q_{\text{b}} = \frac{Z_{\text{LC}}}{R_b}(C/C_b)^2$ is bolometer quality factor. Thus we can rewrite Eq. 1 to

$$P_{\text{b}}(f) = \frac{(1/2Q_{\text{f}})(1/Q_{\text{b}})}{(\omega/\omega_0 - 1)^2 + (1/2Q_{\text{t}})^2} P_{\text{in}}. \tag{19}$$

II. DRIVEN RESONATOR COUPLED TO SEVERAL BATHS

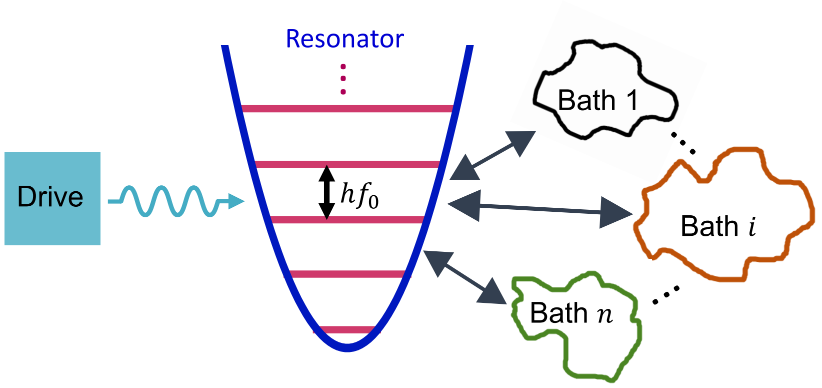

Figure S 2. **Open quantum system picture of the device.** A driven harmonic oscillator coupled to several thermal baths.

In order to anticipate use of the spectrometer for non-linear circuits in the future, we give a short summary on the open quantum system procedure of how to extend the circuit analysis above for systems beyond the linear oscillator discussed here.

The Hamiltonian of the system composed of a driven resonator coupled to various baths, presented in Fig. 2 is given by

$$\mathcal{H} = \mathcal{H}_s + \sum_i \mathcal{H}_i + \mathcal{H}_d + \sum_i \mathcal{H}_{ci}, \tag{20}$$

where $\mathcal{H}_s$ is the Hamiltonian of the resonator, $\mathcal{H}_i$ that of bath i, $\mathcal{H}_d$ denotes the Hamiltonian of the drive, and $\mathcal{H}_{ci}$ is the coupling between the resonator and each bath. The Hamiltonian of the drive (inductive coupling) in the interaction picture reads

$$\mathcal{H}_d = \lambda(t)(\hat{a}\,e^{-i\omega_0 t} + \hat{a}^\dagger\,e^{i\omega_0 t}), \tag{21}$$

where $\hbar\omega_0$ is the energy spacing of the resonator, $\lambda(t) = \Lambda\sin(\Omega t)$ presents the driving term, and $\hat{a}$ and $\hat{a}^\dagger$ are the annihilation and creation operators, respectively. The capacitive coupling of the resonator to a resistive bath $\mathcal{H}_{ci}$ reads

$$\mathcal{H}_{ci} = -i\,g_i(\hat{a}\,e^{-i\omega_0 t} - \hat{a}^\dagger\,e^{i\omega_0 t})v_n(t), \tag{22}$$

where g_i is the coupling constant between the resonator and that bath, and $v_n(t)$ is the noise voltage of the bath.

In the weak coupling regime we derive the general master equation for the reduced density operator ρ of the resonator (in the interaction picture) as

$$\dot{\rho}(t) = \frac{i}{\hbar}[\rho(t), \mathcal{H}_d(t)] + \frac{i}{\hbar}\mathrm{Tr}_\mathrm{B}[\rho_\mathrm{tot}(t), \sum_i \mathcal{H}_{ci}(t)], \tag{23}$$

where $\rho_\mathrm{tot}(t)$ is the total density operator, and Tr_B refers to the trace over the baths. The expression for the power to the bath i is then

$$P_i = \frac{\hbar\omega_0^2}{Q_i}\sum_{r=0}^{\infty}(r+1)\big\{\frac{1}{1-e^{-\beta_i\hbar\omega_0}}\rho_{r+1,r+1} - \frac{1}{e^{\beta_i\hbar\omega_0}-1}\rho_{r,r}\big\}. \tag{24}$$

Ignoring the fast rotating terms, we find the steady-state power that goes to bath i in this case as

$$P_i = \frac{\Lambda^2}{\hbar}\frac{Q_\mathrm{t}^2/Q_i}{1+(2Q_\mathrm{t})^2(\Omega/\omega_0-1)^2} \tag{25}$$

at any temperature and driving level. This result is identical to that from the circuit theory.

III. BOLOMETER CALIBRATION

In this section we describe the temperature calibration of the bolometer. The calibration is performed to obtain the conversion between voltage V_th and its corresponding electronic temperature T_b. The setup is shown in Fig. 3a. At various mixing chamber temperatures ($T_0 = 50 - 400$ mK), a heater voltage (V_h) is applied across a NIS junction (left side) with a tunnel resistance $R_\mathrm{t} \sim 9.43$ kΩ, and V_th is measured across a pair of NIS junctions (right side) with applied bias current $I_\mathrm{b} = 160$ pA. Due to quasiparticle tunneling, the electronic temperature in the normal metal (T_b) is heated or cooled depending on V_h [3, 4] as shown in Fig 3b for few mixing chamber temperatures. Figure 3c shows the V_th at $V_\mathrm{h} = 0$V at different mixing chamber temperatures. We fit the measurement data (blue dots) with a linear function (red line)

$$V_\mathrm{th} = aT_0 + b \tag{26}$$

where $a = -8.144.10^{-4}$ and $b = 4.464.10^{-4}$. The black line is plot obtained from quasiparticle current of a NIS junction with the measured experimental parameters

$$I = \frac{1}{2eR_\mathrm{t}}\int_{-\infty}^{\infty} dE n_\mathrm{S}(E)[f_\mathrm{N}(E-eV) - f_\mathrm{N}(E+eV)] \tag{27}$$

where Fermi distribution is $f_N = 1/(1+e^{E/k_BT_b})$ and the density of states of BCS superconductor is $n_\mathrm{s}(E) = |\mathrm{Re}[(E/\Delta+id)/\sqrt{(E/\Delta+id)^2-1}]|$. Here, superconducting gap of Al film (Δ), Dynes parameter (d), total tunnel resistance of SINIS (R_t) are $\Delta \sim 232$ μeV, $d \sim 2.4$ x 10^{-3}, and $R_\mathrm{t} \sim 22.24$ kΩ, which are obtained from measurements. Electronic temperature T_b saturates around 130 mK due to parasitic heating load from environment P_e, the yellow dashed line is linear model taking into account non-zero P_e. The effective background power P_e varies on cryostat temperatures, at T_0=50 mK, P_e is around 2 fW.

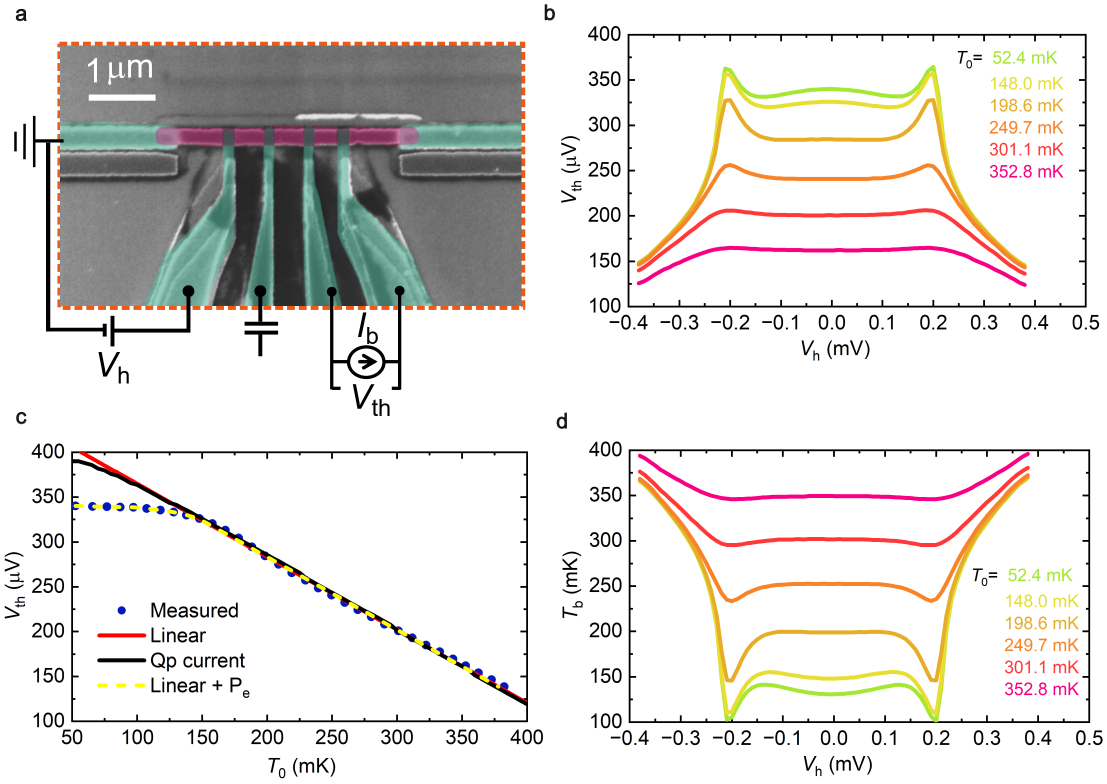

Figure S 3. **Calibration of the bolometer**. **a**, The bolometer setup. **b**, $V_{\rm th}$ versus $V_{\rm h}$ at few diferent cryostat temperatures T_0. **c**, The $V_{\rm th}$ at $V_{\rm h} = 0$ versus temperature of the mixing chamber. The blue dots are the measurement, the red line is linear model, the black line is model based on quasiparticle current formula, and the yellow dashed line is linear model taking into account parasitic heating with power $P_{\rm e}$. **d**, Converted electronic temperature $T_{\rm b}$ versus $V_{\rm h}$.

IV. CONVERSION BETWEEN THE ABSORBED POWER AND THE AVERAGE PHOTON NUMBER

The power $P_{\rm b}$ delivered from the resonator into the bolometer resistor has the form of a rate equation

$$P_{\rm b} = \Gamma_{\rm b}\hbar\omega_0 \left[\langle a^\dagger a\rangle[1 + n_{\rm b}(\hbar\omega_0\beta_{\rm b})] - [1 + \langle a^\dagger a\rangle]n_{\rm b}(\hbar\omega_0\beta_{\rm b})\right] = \Gamma_{\rm b}\hbar\omega_0[\langle a^\dagger a\rangle - n_{\rm b}(\hbar\omega_0\beta_{\rm b})], \qquad (28)$$

where $\Gamma_{\rm b}$ is the zero temperature relaxation rate from resonator into the bolometer ($\Gamma_{\rm b} = 2\pi\gamma_{\rm b}$), $\hbar\omega_0$ is the resonator energy and $n_{\rm b}(\hbar\omega_0\beta_{\rm b})$ is the Bose-Einstein distribution of the bolometer resistor at the resonator energy, $n_{\rm b} = 1/(e^{\hbar\omega_0\beta_{\rm b}} - 1)$ where $\beta_{\rm b} = 1/k_{\rm B}T_{\rm b}$. The average photon number $N \equiv \langle a^\dagger a\rangle$ of the resonator is thus simply converted into absorbed power with the formula

$$N \equiv \langle a^\dagger a\rangle = \frac{P_{\rm b}}{\Gamma_{\rm b}\hbar\omega_0} + n_{\rm b}(\hbar\omega_0\beta_{\rm b}) = \frac{P_{\rm b}Q_{\rm b}}{2\pi h f_0^2} + n_{\rm b}(hf_0\beta_{\rm b}), \qquad (29)$$

where we have written the final expression in terms of measured resonance frequency $f_0 = \omega_0/2\pi$ and the quality factor $Q_{\rm b} = 2\pi f_0/\Gamma_{\rm b}$. The first term is proportional to the input power and therefore, with a sufficiently high input power, the thermal contribution $n_{\rm b}(hf_0\beta_{\rm b})$ can be neglected. In our setup with $f_0 \approx 7.026$ GHz and $T_{\rm b} \approx 130$ mK, we have $n_{\rm b}(hf_0\beta_{\rm b}) \approx 0.07$, which is a negligible correction to the more simple formula $N = \frac{P_{\rm b}Q_{\rm b}}{2\pi h f_0^2}$ as written in the main text.

V. THERMAL SPECTROSCOPY OF RESONATOR-QUBIT

Here we present result of the application of the thermal spectrometer to characterize interaction between resonator and flux qubit. We perform one-tone spectroscopy with the thermal spectrometer and RF spectrometer. The power used is $P_{\rm in} = -120$ dBm and it corresponds to the average photon number $N \sim 0.6$. The device and setups are as

described in the main text. At zero external magnetic flux (Φ_{ext}=0), the flux qubit [5, 6] is inactive since its frequency is much higher than the resonator frequency $f_0 \approx 7.026$ GHz. We sweep the flux around $\Phi_{\text{ext}} \approx \Phi_0/2$, where Φ_0 is the flux quanta, and measure V_{th} and S_{21} at the same time. Figure 4a shows the measured V_{th}. Toward $\Phi_{\text{ext}} = \Phi_0/2$ the resonance shifts and Rabi splitting is observed at $\Phi_{\text{ext}} = 0.4875\Phi_0$. The dispersive shift at $\Phi_0/2$ is observed to be $\chi \sim 44$ MHz. Figure 4b displays results obtained from the RF S_{21} measurement, showing similar spectrum as obtained from the thermal spectrometer.

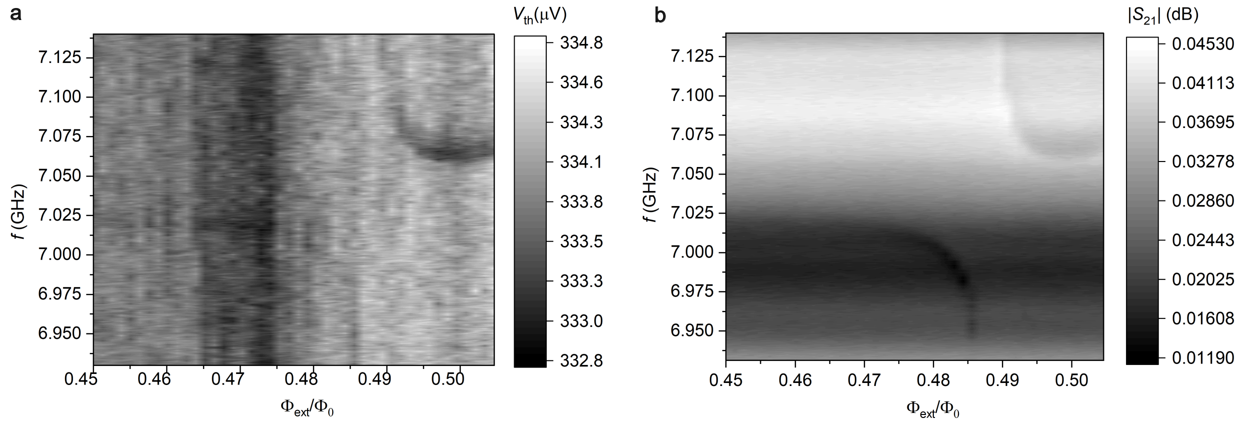

Figure S 4. **Spectroscopy of resonator-qubit**. One-tone spectroscopy measured at low average photon number ($N \sim 0.6$) obtained from: **a**, thermal spectrometer and **b**, RF spectrometer.

[1] D. M. Pozar, *Microwave engineering; 3rd ed.* (Wiley, Hoboken, NJ, 2005).

[2] C. D. Satrya, A. Guthrie, I. K. Mäkinen, and J. P. Pekola, Electromagnetic simulation and microwave circuit approach of heat transport in superconducting qubits, Journal of Physics Communications **7**, 015005 (2023).

[3] F. Giazotto, T. T. Heikkilä, A. Luukanen, A. M. Savin, and J. P. Pekola, Opportunities for mesoscopics in thermometry and refrigeration: Physics and applications, Rev. Mod. Phys. **78**, 217 (2006).

[4] M. Nahum, T. M. Eiles, and J. M. Martinis, Electronic microrefrigerator based on a normal-insulator-superconductor tunnel junction, Applied Physics Letters **65**, 3123 (1994).

[5] R. Upadhyay, G. Thomas, Y.-C. Chang, D. S. Golubev, A. Guthrie, A. Gubaydullin, J. T. Peltonen, and J. P. Pekola, Robust strong-coupling architecture in circuit quantum electrodynamics, Physical review applied **16**, 10.1103/physrevapplied.16.044045 (2021).

[6] A. A. Abdumalikov, O. Astafiev, Y. Nakamura, Y. A. Pashkin, and J. Tsai, Vacuum rabi splitting due to strong coupling of a flux qubit and a coplanar-waveguide resonator, Phys. Rev. B **78**, 180502 (2008).
